# Arms-Based Meta-Analysis of Microbiological Endpoints of 88 VAP Prevention Studies Using Antimicrobial Versus Non-Antimicrobial Strategies—Towards ‘VAP-Zero’?

**DOI:** 10.3390/antibiotics15020221

**Published:** 2026-02-17

**Authors:** James C. Hurley

**Affiliations:** 1 Melbourne Medical School, University of Melbourne, Parkville 3010, Australia; hurleyjc@unimelb.edu.au or jamesh@gh.org.au; 2 Ballarat Health Services, Grampians Health, Ballarat Central 3350, Australia; 3 Ballarat Clinical School, Deakin University, Ballarat Central 3350, Australia

**Keywords:** antibiotic-based decontamination, selective digestive decontamination, VAP-zero, intensive care unit, Ventilator-associated pneumonia, *Staphylococcus aureus*, *Pseudomonas aeruginosa*, *Acinetobacter* species

## Abstract

**Background/Objectives:** In traditional contrast-based meta-analyses of randomized concurrent controlled trials (RCCTs), topical antibiotic prophylaxis (TAP) appears more effective than either antiseptic-based or non-antimicrobial-based interventions for preventing ventilator-associated pneumonia (VAP). The objective here is to use arm-based methods to determine whether this effectiveness translates towards achieving VAP-zero, both overall and specifically for VAP in association with *Staphylococcus aureus*, *Pseudomonas aeruginosa*, and *Acinetobacter* species among the same RCCTs. **Methods**: Data were extracted from RCCTs sourced primarily from Cochrane reviews of VAP prevention interventions. Arms-based and contrast-based methods of meta-analyses of the VAP prevention effect size and the VAP incidence per 100 patients receiving mechanical ventilation were obtained using random effects methods. **Results**: The VAP prevention intervention effect sizes derived by contrast-based versus arms-based meta-analyses were similar for each of the three broad types of interventions. The overall VAP prevention effect of antibiotic-based interventions by contrast-based and arms-based methods were 0.39 (95% confidence interval 0.33 to 0.46; n = 28) versus 0.39 (95% confidence interval 0.32 to 0.47; n = 28), respectively. Surprisingly, the arms-based analysis revealed that the summary VAP incidence, both overall and for each of *Staphylococcus aureus*, *Pseudomonas aeruginosa*, and *Acinetobacter* species within antibiotic intervention groups, were similar to the respective summary incidences within intervention groups of non-antimicrobial RCCTs. **Conclusions**: VAP-zero, both overall and in association with specific microbial sub-types, has remained elusive using antimicrobial-based interventions. This inference was not evident from a contrast-based analysis.

## 1. Introduction

There are numerous randomized concurrent controlled trials of interventions to prevent the occurrence of ventilator-associated pneumonia (VAP) [1,2,3,4,5,6,7,8,9,10,11,12,13,14,15,16,17,18,19,20,21,22,23,24,25,26,27,28,29,30,31,32,33,34,35,36,37,38,39,40,41,42,43,44,45,46,47,48,49,50,51,52,53,54,55,56,57,58,59,60,61,62,63,64,65,66,67,68,69,70,71,72,73,74,75,76,77,78,79,80,81,82,83,84,85,86,87,88]. The interventions can be broadly classified into non-antimicrobial- and antimicrobial-based interventions. The latter include antiseptic-based decontamination and antibiotic-based decontamination interventions. In total, 88 randomized concurrent controlled trials present data for VAP isolates including *Staphylococcus aureus*, *Pseudomonas aeruginosa*, and *Acinetobacter* species (Table 1).

In preventing VAP, an aspirational goal for many intensive care units is to approach VAP-zero, an aspiration in line with the VAP-zero initiative [101]. On the other hand, a contrary view is that VAP is inevitable, especially when considering ICU patient populations with greater lengths of stay [102,103,104,105].

Antimicrobial-based methods appear more effective than non-antimicrobial-based methods in traditional contrast-based meta-analyses, but these methods are unable to indicate the propensity of the intervention groups of the various RCCTs to approach VAP-zero. Such an appraisal would require each control and intervention arm of the randomized concurrent controlled trials analyzed separately in an arms-based meta-analysis. Arms-based meta-analysis can most simply be undertaken using methods applied to the analysis of diagnostic tests such as Summary Receiver Operator Characteristic (SROC) plots [106,107,108].

VAP occurs in 5 to 40% of patients undergoing mechanical ventilation in intensive care units [109,110,111,112,113,114,115]. It would be expected that the effect of non-antimicrobial-based interventions would be more similar in their prevention effect versus each of *Staphylococcus aureus*, *Pseudomonas aeruginosa*, and *Acinetobacter* species than would be the case for antimicrobial-based interventions. However, the impact of the various non-antimicrobial-based versus antimicrobial-based interventions on the occurrence of VAP in association with these various types of isolates is not clear. Specifically, might ‘VAP-zero’ be more readily attainable for VAP in association with some isolates rather than others and with one broad category of intervention? A contrast-based analysis cannot address this question.

There are four objectives here: first, to compare the VAP prevention effect size estimates of various prevention interventions within the literature as generated using contrast-based versus arms-based methods of meta-analysis; second, to triangulate the estimates here with the previous effect size summaries for non-antimicrobial-, antiseptic- and antibiotic-based interventions within the literature; third, to then also compare the prevention effect size estimates for VAP in association with *Staphylococcus aureus*, *Pseudomonas aeruginosa*, and *Acinetobacter* species; finally, using the arms-based approach including a meta-regression, compare the different interventions towards their propensity to attain VAP-zero.

## 2. Materials and Methods

### 2.1. Study Selection and Decant of Groups

**Figure 1:**
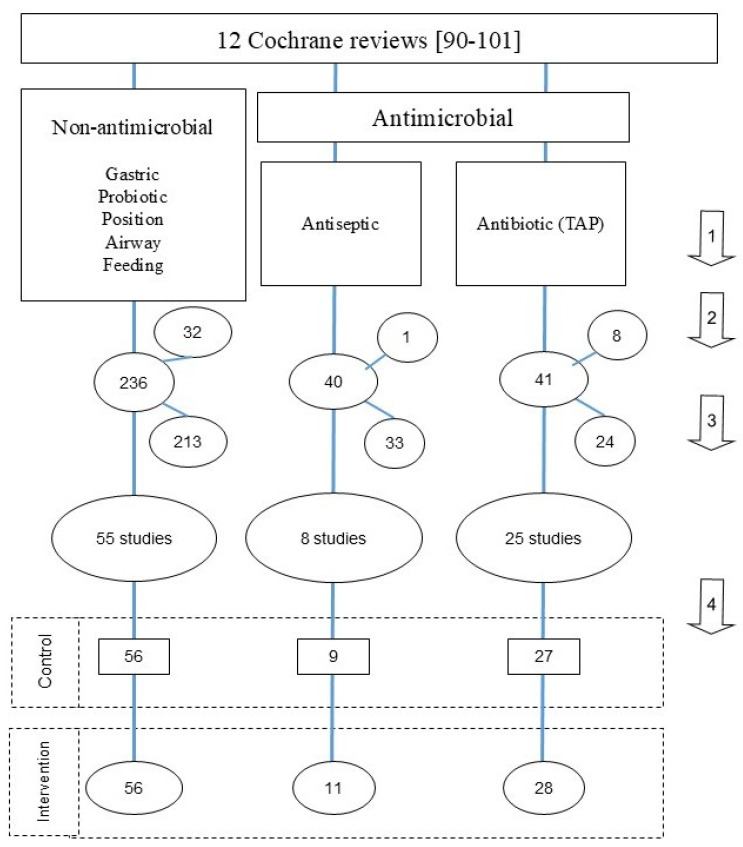
Flow diagram of study selection. The four steps are as follows. 1. An electronic search of the Cochrane database [90,91,92,93,94,95,96,97,98,99,100,101] for systematic reviews of non-antimicrobial [90,91,92,93,94,95,96,97], antiseptic [98,99] and antibiotic [100,101] interventions to prevent VAP among MV patients in the ICU up to November 2025. 2. Additional studies meeting the inclusion criteria obtained from Google Scholar. The studies were streamed into three categories of RCCT; non-antimicrobial [1,2,3,4,5,6,7,8,9,10,11,12,13,14,15,16,17,18,19,20,21,22,23,24,25,26,27,28,29,30,31,32,33,34,35,36,37,38,39,40,41,42,43,44,45,46,47,48,49,50,51,52,53,54,55], antiseptic [56,57,58,59,60,61,62,63] and antibiotic [64,65,66,67,68,69,70,71,72,73,74,75,76,77,78,79,80,81,82,83,84,85,86,87,88]. 3. Exclusion criteria: n < 25; lacking VAP data; fewer than two VAP isolate types listed; publication prior to 1985; patient population selected for candidemia risk factors. 4. Control and intervention groups were decanted from the RCCT’s.

The literature search used here (Figure 1) is as described previously [116,117]. Cochrane reviews and other systematic reviews [118,119,120,121,122,123] were used as the primary source of studies, with additional studies being found by snowball sampling using the “Related articles” function within Google Scholar. Clinical trial databases were not accessed.

The inclusion criteria were cohorts of patients requiring prolonged (>24 h) ICU stays for which the incidence proportions of VAP overall and VAP in association with *Staphylococcus aureus*, *Pseudomonas aeruginosa*, and *Acinetobacter* species. The listing of at least two of the three infections was required so as to avoid selecting studies which may have reported only the most prominent VAP isolate type. Data were extracted for each component group where there was more than one type of control or intervention group.

The studies were classified into three broad groups of study interventions being non- antimicrobial, antiseptic-based, and antibiotic-based. Non-antimicrobial interventions were studies of various approaches to the control of upper gastrointestinal tract colonization through various stress ulcer prevention or feeding approaches, as well as various approaches to control airway colonization through airway management [90,91,92,93,94,95].

Antiseptic-based interventions included the use of agents such as chlorhexidine, povidone-iodine and iseganan. All antiseptic exposures were included regardless of whether the application was to the oropharynx, by toothbrushing, or by body wash [98,99]. Antibiotic-based interventions included the use of either topical antibiotic prophylaxis (TAP) to the oropharynx or stomach (without regard to the specific antibiotic constituents) or whether protocolized parenteral antibiotic prophylaxis (PPAP) was used in addition to the topical antibiotics or used alone [100,101].

The inclusion criteria were deliberately broad without regard to the frequency or duration of interventions under study or any criteria of study quality. Studies published between 1985 and 2024 were included. Studies originating from pediatric ICUs were not excluded. Studies for which patient inclusion was on the basis of risk factors for fungal infections and studies with fewer than 25 patients were excluded.

### 2.2. Outcomes of Interest

Regarding the count of VAP, both overall and in association with each of *Staphylococcus aureus*, *Pseudomonas aeruginosa*, and *Acinetobacter* species, there was no imputation of missing data. Study quality was taken as judged in each source document and standardized into a simple binary score based on whether the quality score was assigned a majority of possible scoring points.

The independent variable in the regression models was the mean length of the ICU stay (LOS). If this was not available, the median LOS or the mean or median duration of mechanical ventilation were used. The VAP incidence proportion and LOS data were all derived from the original publications. The VAP incidence proportion, being count data, were logit transformed using the number receiving prolonged (>24 h) mechanical ventilation as the denominator. The LOS data were positively skewed and were log transformed.

### 2.3. Summary Effect Size; Contrast-Based Analysis

Indicative summary prevention effect sizes versus VAP, for each category, were derived from all studies. Summary prevention effect sizes versus VAP in association with *Staphylococcus aureus*, *Pseudomonas aeruginosa*, and *Acinetobacter* species were also derived where available.

Study specific and overall summary VAP prevention effect sizes and associated 95% confidence interval were calculated for each category using random effect methods of meta-analysis. The data were set for two group comparisons of binary outcomes using the ‘meta’ command in Stata 18 (Stata Corp.; College Station, TX, USA) and the default restricted maximum likelihood [124].

### 2.4. Arms-Based Analysis and SROC Plots

The data from the component control and intervention groups of randomized concurrent controlled trials were decanted from each randomized concurrent controlled trial with care to include the groups from the three arm studies only once. Summary VAP incidence proportion data were derived using the ‘meta’ command in Stata 18 and the default restricted maximum likelihood. SROC plots, being a bivariate plot of control versus intervention group incidences of VAP, were derived [106,107].

### 2.5. Arms-Based Meta-Regression

The relationship between study specific prevention effect sizes versus log transformed LOS of each of the three broad categories of intervention toward the prevention of VAP were modelled by meta-regression.

### 2.6. Availability of Data and Materials

All data generated or analyzed during this study have been included in this published article (see Table 1).

## 3. Results

### 3.1. Characteristics of the Studies

Of the 88 randomized concurrent controlled trials identified by the search, 48 studies were found in either twelve Cochrane [90,91,92,93,94,95,96,97,98,99,100,101] or other systematic reviews [102,103,104,105,106,107,108,109,110,111,112,113,114,115,116,117,118,119,120,121,122,123,125]. Most studies were published between 1990 and 2010, and most had a mean ICU LOS exceeding ten days. Five randomized concurrent controlled trials had either more than one type of intervention group or more than one type of control group. Most groups had between 50 and 100 patients. Most studies originated from either North American or European ICUs. VAP count data for all three VAP isolates were available from 58 randomized concurrent controlled trials (Table 2).

The most common non-antimicrobial interventions were gastric pH management (10 studies), heat and moisture exchange (11 studies) and endotracheal tube management (8 studies). The most common antiseptic intervention was topical chlorhexidine (4 studies), and the most common antibiotic intervention was some type of combination topical antibiotic and protocolized parenteral antibiotic prophylaxis regimen (11 studies).

### 3.2. Prevention Effect Sizes

The study specific and summary effect sizes derived by contrast-based and arms-based methods for the three categories of intervention against VAP are presented in Table 3. Significant summary prevention effects against both overall VAP and *S. aureus* VAP were evident for all three categories. There were significant summary prevention effects against *Pseudomonas* VAP for the non-antimicrobial and antibiotic categories.

The summary effect size estimates derived by arms-based methods, as diagnostic odds ratios, were similar to those derived by contrast-based methods for overall VAP, *Pseudomonas* VAP, and *S. aureus* VAP. The SROC models failed to converge with the *Acinetobacter* for the non-antimicrobial and antiseptic models.

**Figure 2:**
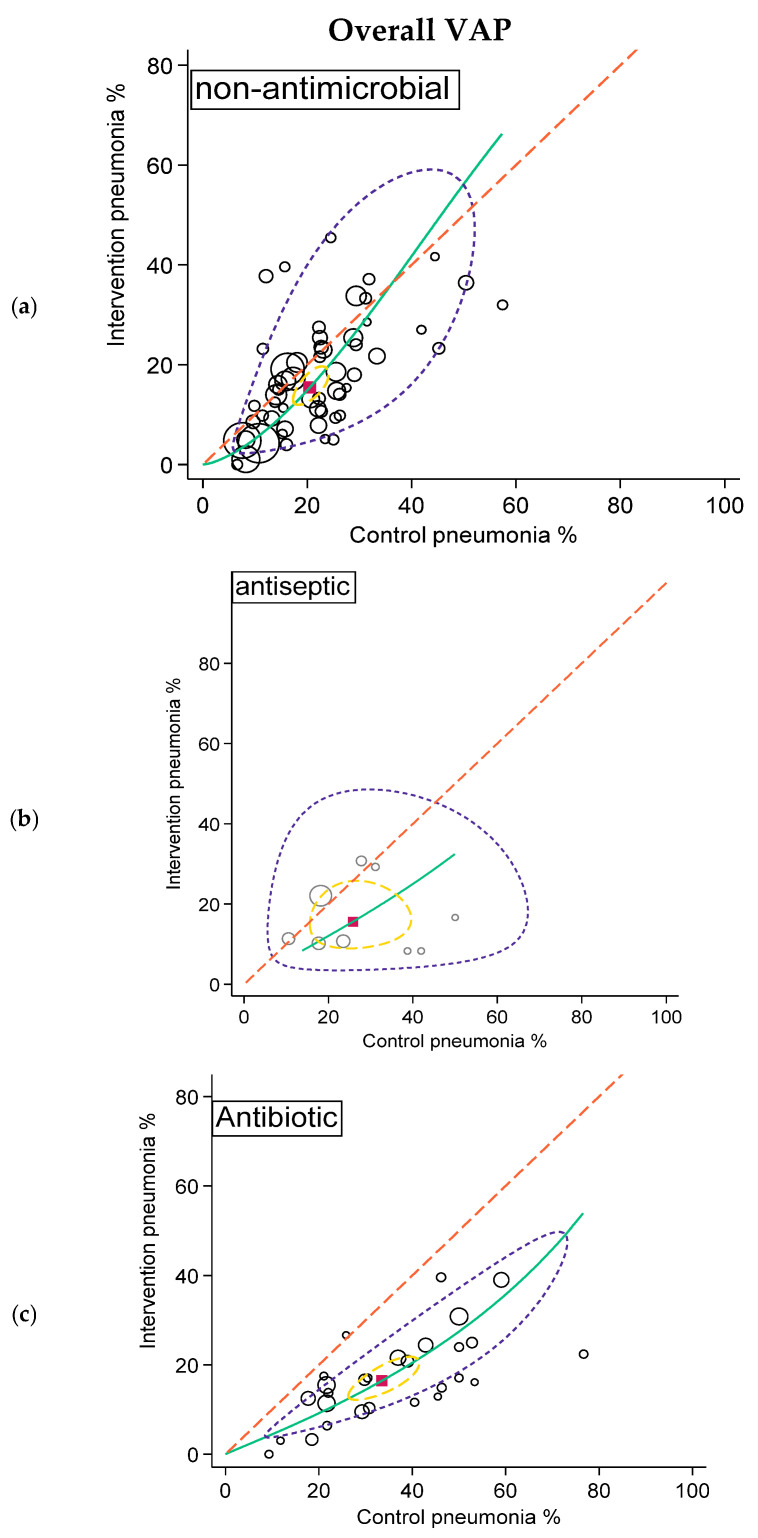
SROC plots of summary effect size of non-antimicrobial (**a**; **top**), antiseptic (**b**; **middle**), and antibiotic (**c**; **bottom**) interventions in preventing overall VAP. Pneumonia incidence among control and intervention groups with symbol size proportional to group size (hollow circles). The diagonal dotted line is the line of equivalence and the curved green line is the summary SROC curve. Also shown are the summary point (solid red square), the hierarchical summary ROC curve (green) with 95% confidence limits (dotted yellow inner ellipse), and 95% prediction limits (dotted purple outer ellipse).

**Figure 3:**
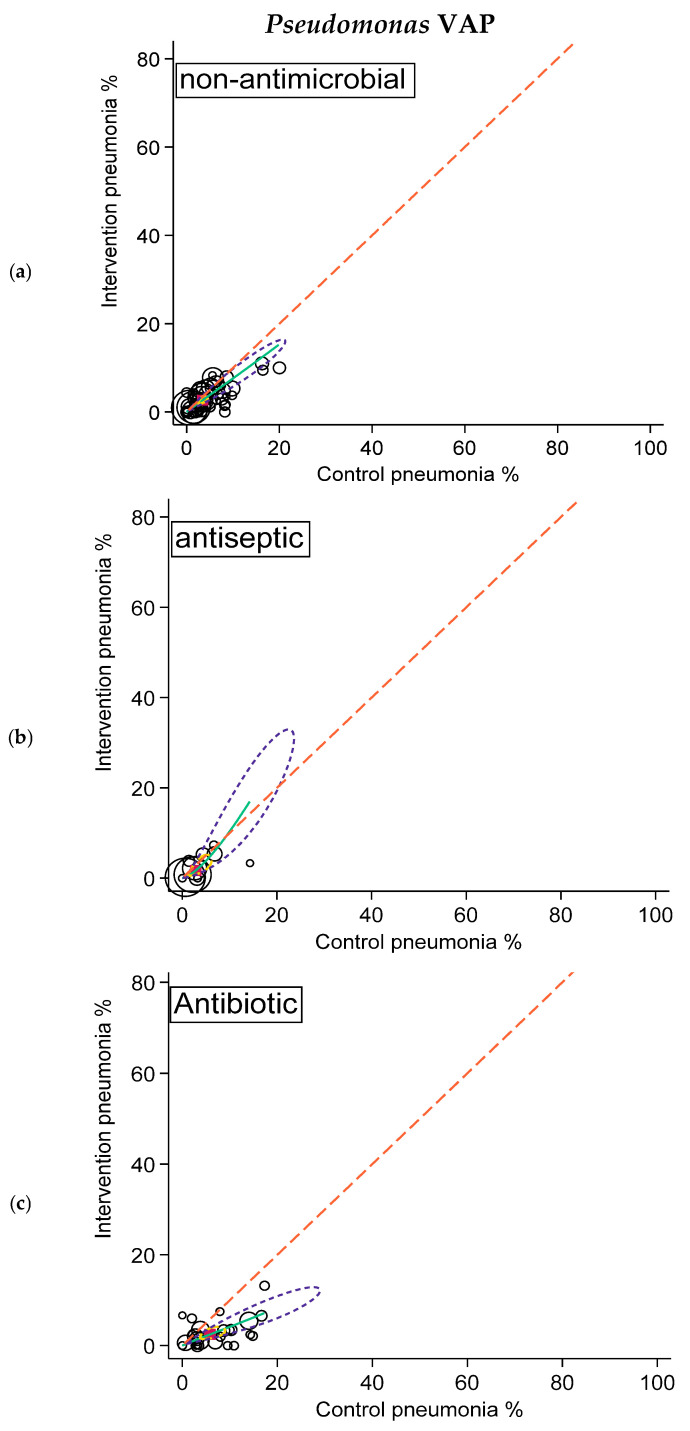
SROC plots of summary effect size of non-antimicrobial (**a**; **top**), antiseptic (**b**; **middle**), and antibiotic (**c**; **bottom**) interventions in preventing *Pseudomonas* VAP. Pneumonia incidence among control and intervention groups with symbol size proportional to group size (hollow circles). The diagonal dotted line is the line of equivalence and the curved green line is the summary SROC curve. Also shown are the summary point (solid red square), the hierarchical summary ROC curve (green) with 95% confidence limits (dotted yellow inner ellipse), and 95% prediction limits (dotted purple outer ellipse).

**Figure 4:**
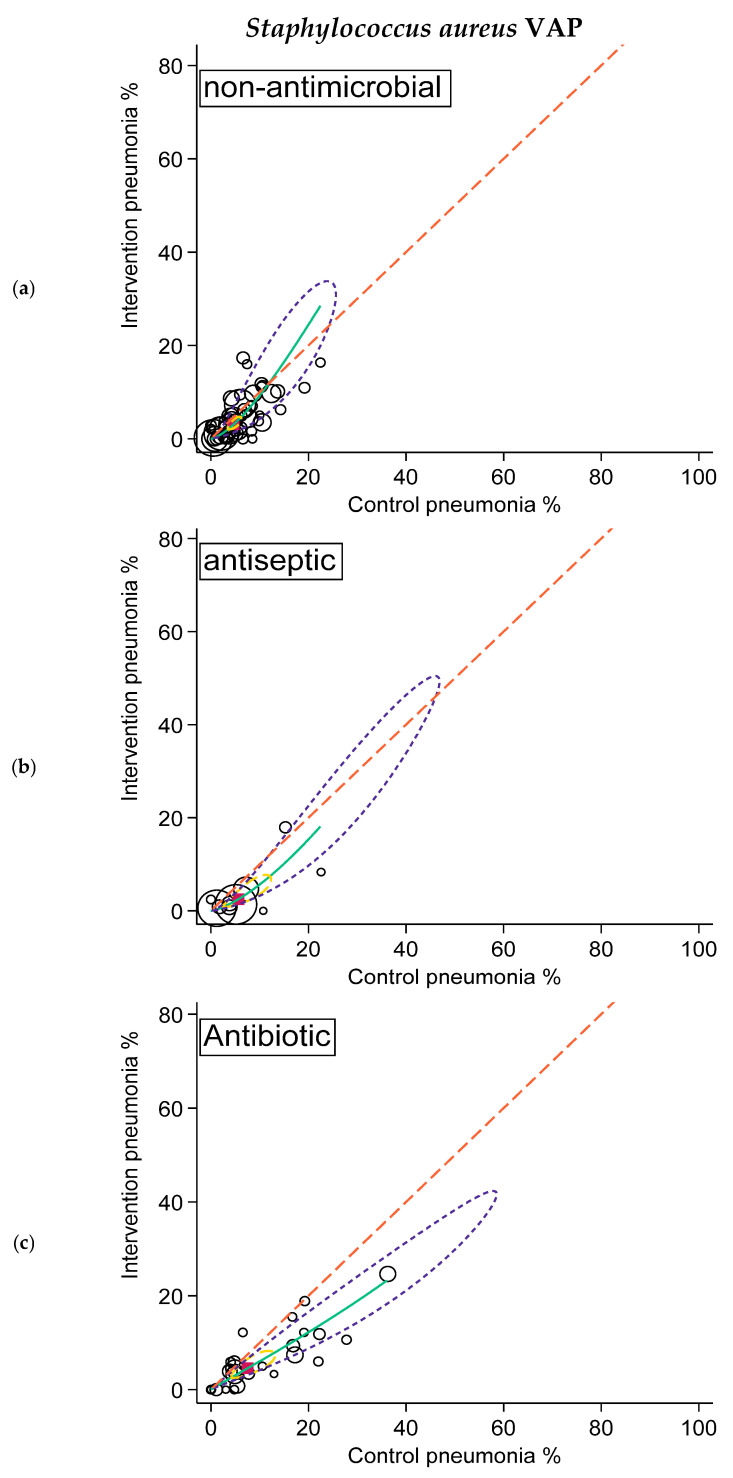
SROC plots of summary effect size (and associated 95% confidence and 95% prediction ellipses) of non-antimicrobial (**a**; **top**), antiseptic (**b**; **middle**), and antibiotic (**c**; **bottom**) interventions in preventing *Staphylococcus aureus* VAP. The diagonal dotted line is the line of equivalence and the curved green line is the summary SROC curve. Also shown are the summary point (solid red square), the hierarchical summary ROC curve (green) with 95% confidence limits (dotted yellow inner ellipse), and 95% prediction limits (dotted purple outer ellipse).

### 3.3. Arms-Based Analysis and Meta-Regression

The summary overall VAP incidences for the control groups of the studies of antibiotic interventions, being 34%, were higher versus the summary incidences for the control groups for the other two categories, being 21 and 22%. Paradoxically, the summary overall VAP incidences for the intervention groups, being in the range of 12 to 16%, were similar across the three categories (Table 3).

The mean overall VAP proportion was above 40% for 11 of 27 antibiotic control groups but only six of the 63 control groups in the other studies. The mean overall VAP proportion was below 5% for only 11 intervention groups, eight from non-antimicrobial studies and three from studies of antibiotic-based interventions (Figure 2).

Likewise, the summary *Pseudomonas* VAP and *Staphylococcus aureus* VAP incidences for the control groups of the studies of antibiotic interventions were higher versus the summary VAP incidences for the control groups of the other two categories. Again, paradoxically, the summary VAP incidences for these isolates for the intervention groups for the three categories were more similar to each other (Table 3; Figure 3 and Figure 4).

These paradoxical VAP incidences were also apparent in the arms-based meta-regression whether with or without adjustment for LOS (Table 4). Membership in a concurrent control group of a study of an antibiotic-based intervention was associated with a significantly higher incidence of all types of VAP. Membership in an intervention group of a study of an antibiotic-based intervention was not associated with a lower incidence of overall VAP, although it was associated with a lower incidence of *Pseudomonas* VAP in the adjusted model.

## 4. Discussion

With respect to the four objectives here, firstly, the effect size estimates for three broad categories of prevention interventions against overall VAP derived using contrast-based versus arms-based meta-analysis methods here were substantially equivalent.

Secondly, these prevention effect size estimates triangulated with previous effect size summaries for the three broad categories of prevention interventions derived for a larger number of studies within the Cochrane reviews and other literature sources (Table 5). The summary prevention effect sizes of the various non-antimicrobial interventions derived for a larger number of studies within the literature generally had risk ratios between 0.5 and 0.95, which compares to the odds ratio derived here from all 56 RCCTs of non-antimicrobial interventions of 0.73 (95% confidence interval 0.61 to 0.86).

Third, the prevention effect estimates for the various interventions differed slightly in their prevention effect towards VAP associated with each of *Staphylococcus aureus*, *Pseudomonas aeruginosa*, and *Acinetobacter* species. However, with each, the odds ratios derived from contrast-based versus arms-based meta-analysis methods were substantially similar (Table 3). Moreover, the estimates obtained in the meta-regression models were robust to adjustment for group mean LOS, quality score, and year of publication (Table 4). In all models, membership in a concurrent control group of an RCCT of an antibiotic-based VAP prevention intervention were strong and positive predictors for each of the associated VAP incidences; by contrast, membership in an intervention group of an RCCT of an antibiotic-based VAP prevention intervention were weaker and generally insignificant negative predictors.

Finally, in the arms-based analysis and SROC plots, VAP-zero appeared to be elusive for both VAP overall and VAP in association with each of *Staphylococcus aureus*, *Pseudomonas aeruginosa*, and *Acinetobacter* species. For example, incidences for overall VAP of less than 5%, the lower limit of the expert VAP incidence range, were rare (Figure 2). Moreover, low VAP incidences were no more common among studies of antimicrobial versus non-antimicrobial interventions either for overall VAP or for VAP with any of the specific microbial types.

The mean VAP incidence and mean *Pseudomonas aeruginosa*-associated VAP incidence estimated here were similar to that observed in a worldwide multi-center prospective study [127].

The strengths in the analysis here included the large number of studies reporting at least two VAP types. Studies with only one VAP type might have either reported only the most prominent VAP type or the study may have had a specific focus on that VAP type [128]. This may have created a potential reporting bias.

The principle underlying contrast-based methods is that the random assignment of intervention, which is central to causal inference from RCCTs, is retained in the meta-analysis model [129]. Hence, the fundamental criticism of arms-based methods was that it ‘breaks’ this random assignment [130]. On the other hand, if there was spillover, the stable unit treatment value assumption, which was central to causal inference originating from random assignment, is no longer tenable. In which case, an arms-based analysis is to be preferred [131]. Moreover, arms-based analysis can enable comparisons of event rates observed within each arm to external benchmarks, such as the expert opinion VAP incidence range, towards deriving population level inferences.

The analysis extended the novel use of SROC methods as an arms-based approach to the analysis of RCCT data [107,108]. The SROC method was recently adapted for application to the meta-analysis of diagnostic tests. The SROC plot resembled the L’abbe plot as derived within a meta-analysis of RCCTs. Each displayed the dispersion in event rates in the two component groups along the *y*-axis for one versus the *x*-axis for the other [124]. For the L’abbe plot, these were the event rates in the intervention versus control groups, respectively. For the SROC plot, these were the test positive rates among the diseased (sensitivity) versus the non-diseased (which equates to 1 minus specificity), respectively. In both cases, the diagonal (y = x line) represented the locus where the event rates in the two populations in the comparison were equal. The two plots differed in how the covariation away from this line was displayed and how event rate dispersion was inferred. For the L’abbe plot, depending on whether the ES was defined as an odds ratio (OR), a risk ratio (RR), or a risk difference (RD), this gave a visual representation of covariation as either a line parallel to the y = x line (RD), a line that passed through the origin (RR), or a curve (OR), respectively. For the L’abbe plot, dispersion was merely a subjective visual inference which was dependent on whether the presumptive underlying relationship was an RD, RR, or OR.

For the SROC plot, on the other hand, the underlying relationship was always displayed as an OR and the dispersion in event rates, quantified as a summary point together with an enveloping 95% prediction ellipse, enabling projections of the sensitivity and specificity that future applications of the diagnostic test of interest might experience. The SROC displayed the summary operating curve which mapped the summary values of sensitivity and specificity within the SROC plot. Moreover, instead of two unidirectional 95% confidence limits, these models provided bi-directional 95% confidence regions (as ellipses) rather than as together with 95% prediction ellipses [108].

By displaying the event rates in both the control and intervention arms, arms-based methods can accommodate the potential issue of spillover effects to concurrent patients within the ICU not receiving decontamination as a possible ‘driver’ of the whole of intensive care unit infection event rates [107].

Antimicrobial-based interventions, using either topical antiseptics and oral care [98,99] or antibiotics [100,101], were presumed to alter the microbiome of the entire ICU. This spillover of intervention effect was anticipated from the first study [132] being postulated as “*…having heavily contaminated patients next to decontaminated patients might adversely affect the potentially beneficial results* [postulate one]. *Secondly, a reduction of the number of contagious patients by applying [selective digestive decontamination] SDD in half of them, might reduce the acquisition, colonisation and infection incidence in the not-SDD-treated control group* [postulate two]” [132].

Surprisingly, the randomized controlled trials of antimicrobial-based decontamination interventions with concurrent controls had an overall VAP incidence which was higher, not lower as was postulated above, than what might be expected in comparison to expert VAP incidence range estimates [109,110,111,112,113,114,115]. This high VAP remains unexplained. These RCCTs also had higher incidences of blood stream infections [102], candidemia [114], and mortality [133] which likewise are unexplained.

Rebound infection on withdrawal of antibiotic-based infection prevention interventions also need to be considered. Rebound infection had been noted among patients that became neutropenic following cytotoxic chemotherapy in hematology units in the 1970s. These severe and occasionally fatal infections were observed in patients who had prematurely discontinued the antibiotic-based intervention due to its intolerable taste. Rebound sepsis has been noted following hospital discharge among patients exposed to antibiotic therapy considered high risk for causing microbiome disruption [134].

Rebound may be imperceptible without specific surveillance for colonization and infections on withdrawal of decontamination interventions. Rebound following antibiotic-based discontinuation and ICU discharge manifested as a 50% increased risk of hospital-acquired infection [135]. Rebound of ceftazidime resistant Gram-negative bacteria may occur as a ‘whole of ICU’ phenomenon not limited to the antibiotic-based recipients, persisting as an ecological effect for several months after antibiotic-based withdrawal [136,137].

The use of protocolized parenteral antibiotic prophylaxis within some concurrent control groups may have modified the rebound and spillover effects from the intervention groups within these RCCTs.

There was an uncertain amount of spillover effect in these concurrent controlled RCCTs of VAP prevention using antimicrobial-based interventions which was as previously noted for several end points [138,139]. Any spillover effect would conflate the apparent prevention effect [140] which underlie the paradoxical observations [141].

### Limitations

Several limitations should be considered.

There was considerable heterogeneity in the interventions, populations, study quality and RCCT designs among studies published over several decades included in the analysis here with no ability to adjust for underlying patient risk. The definitions of VAP used in various studies varied and this further added to the heterogeneity for endpoints related to VAP incidence. Despite this heterogeneity in overall VAP incidences, incidences < 5%, the lower limit of the expert VAP incidence range, were rare.

Whilst the RCCTs included here generally rated highly for study quality within Cochrane reviews, the potential effects of spillover and rebound were not recognized in these quality scores. Hence, the prevention effect estimates were considered ‘indicative’, and primarily related to the population level rather than the patient level of analysis.

The literature search has been opportunistic rather than systematic. By using existing systematic reviews as a starting point, the key interventions from the broadly selected studies can be readily identified and classified. These Cochrane reviews served as a source of effect size estimates for triangulation.

Mean LOS and even median LOS were crude measures of group level exposure for each group in the ICU context and exposure to the infection prevention interventions in the intervention groups. Of note, even cohorts with short mean LOS will contain patients with long LOS and vice versa. The analysis was ecological, and the estimates related to the impacts of antiseptic-based and antibiotic-based interventions on ICU patient cohorts. The associations for group-wide exposures may not equate to associations at the patient level of exposure.

Many studies of decontamination interventions will have been underpowered to adequately assess key safety end points or to assess for novel microbiome interactions that have been thought to contribute to VAP [116].

There has been no imputation of missing data. There has been no search for data outside of the English-language literature. Only the three major subgroups have been analyzed with no further subgroup analysis due to the limited number of studies.

Finally, there could be the potential for publication and reporting bias if studies merely listed only the most prominent VAP isolate. This bias has been addressed by limiting inclusion to those studies reporting at least two isolates. However, this leaves a substantial number of studies which failed to report the VAP isolates. Also, uncommon isolates that might be causes of VAP have not been considered, such as *Enterococci* [139]. Of note, the effect size metrics for the prevention of overall VAP estimates here were similar to those derived from all available studies whether or not a listing VAP isolate data was available.

## 5. Conclusions

VAP-zero both overall and in association with specific microbial sub-types remains elusive using antimicrobial-based interventions. The control group incidence of VAP in association with *Staphylococcus aureus*, *Acinetobacter*, and *Pseudomonas aeruginosa* was unusually high among RCCTs that showed apparent effectiveness of antimicrobial-based VAP prevention interventions.

## Figures and Tables

**Table 1:** Characteristics of studies.

						Patients	VAP	VAP	VAP Isolate Counts		
Author & Intervention	C/I	Year	Ref.	QS	LOS	(n)	(n)	%	*Pseudomonas*	*Acinetobacter*	*Staph aureus*
**Non-Antimicrobial**											
Acosta-Escribano	C	2010	1	1	18	54	31	57	4	0	4
Acosta-Escribano Feed	I	2010	1	1	16	50	16	32	3	0	8
Bonten	C	1995	2	2	16.8	67	15	22	11	0	4
Bonten Gastric	I	1995	2	2	19	74	16	22	7	1	7
Cook	C	1998	3	2	13.7	604	98	16	21	.	36
Cook Gastric	I	1998	3	2	12.8	596	114	19	20	.	44
Damas	C	2022	4	1	11	155	32	21	4	1	3
Damas ETT	I	2022	4	1	11	168	22	13	2	.	1
Dat	C	2022	5	1	19	301	52	17	17	15	6
Dat ETT	I	2022	5	1	20	296	51	17	23	16	7
Daumal	C	1999	6	1	6.1	174	25	14	11	.	7
Daumal HME	I	1999	6	1	7.3	187	30	16	12	.	9
David	C	2011	7	1	6	100	29	29	20	.	5
David ETT	I	2011	7	1	6	100	18	18	10	.	4
Djedaini	C	1995	8	1	9.7	61	6	10	0	3	0
Djedaini HME	I	1995	8	1	9.3	68	8	12	3	0	2
Drakulovic	C	1999	9	1	9.7	47	11	23	3	1	4
Drakulovic Position	I	1999	9	1	9.3	39	2	5	1	0	0
Dreyfuss	C	1991	10	1	10	35	11	31	3	3	2
Dreyfuss CC	I	1991	10	1	12.8	28	8	29	1	2	1
Dreyfuss	C	1995	11	1	10	70	8	11	1	2	2
Dreyfuss HME	I	1995	11	1	12.5	61	6	10	0	3	0
Driks	C	1987	12	2	14.2	61	7	11	5	.	4
Driks Gastric	I	1987	12	2	10.6	69	16	23	1	.	0
Forestier	C	2008	13	1	13	106	24	23	8	.	11
Forestier Feed	I	2008	13	1	13	102	24	24	3	.	12
Giamarellos-Bourboulis	C	2009	14	1	15	36	16	44	2	12	.
Giamarellos-Bourboulis F	I	2009	14	1	15	36	15	42	3	5	.
Heyland	C	1999	15	2	12	46	7	15	0	.	0
Heyland Feed	I	1999	15	2	13	49	3	6	0	.	1
Holzapfel	C	1999	16	1	14.6	200	51	26	6	3	21
Holzapfel ETT	I	1999	16	1	16.5	199	37	19	10	4	7
Kappstein	C	1991	17	2	5	49	12	24	.	.	11
Kappstein Gastric	I	1991	17	2	5	55	25	45	.	.	9
Kirton	C	1997	18	1	17	140	22	16	6	.	6
Kirton HME	I	1997	18	1	20	140	10	7	6	.	6
Knight	C	2009	19	2	7	129	17	13	1	1	1
Knight Feed	I	2009	19	2	6	130	12	9	0	3	0
Kollef	C	1999	20	2	4	183	15	8	1	.	7
Kollef ETT	I	1999	20	2	4	160	8	5	2	.	1
Kollef	C	2008	21	2	4	743	56	8	11	5	16
Kollef ETT	I	2008	21	2	4	766	37	5	8	1	9
Kortbeek	C	1999	22	1	7	43	18	42	0	1	.
Kortbeek Feed	I	1999	22	1	10	37	10	27	0	1	.
Lacherade	C	2005	23	1	25	184	53	29	14	0	16
Lacherade HME	I	2005	23	1	21	185	47	25	9	2	18
Lacherade	C	2010	24	1	11	164	42	26	16	2	8
Lacherade SSD	I	2010	24	1	11	169	25	15	9	2	2
Laueny	C	2014	25	1	17	91	11	12	0	0	6
Laueny other	I	2014	25	1	11	98	37	38	0	1	17
Lorente	C	2003	26	1	18	116	26	22	10	1	8
Lorente HME	I	2003	26	1	16	114	29	25	9	0	7
Lorente	C	2004	27	1	16.4	143	33	23	8	1	6
Lorente CC	I	2004	27	1	19.8	161	37	23	9	3	14
Lorente	C	2005	28	2	12.7	233	42	18	12	2	11
Lorente TS	I	2005	28	2	12.5	210	43	20	12	1	10
Lorente	C	2006	29	1	9.5	221	31	14	7	1	8
Lorente TS	I	2006	29	1	9.9	236	33	14	9	1	8
Lorente	C	2006	30	1	21	51	8	16	5	.	5
Lorente HME	I	2006	30	1	20	53	21	40	2	.	2
Lorente	C	2007	31	1	15.5	140	31	22	4	1	8
Lorente SSD	I	2007	31	1	14.1	140	11	8	4	3	2
Lorente	C	2014	32	1	16	150	33	22	6	1	5
Lorente SSD	I	2014	32	1	15	134	15	11	3	0	1
Mahmoodpoor	C	2017	33	1	18	138	46	33	3	3	4
Mahmoodpoor ETT	I	2017	33	1	15	138	30	22	2	0	1
Manzano	C	2008	34	1	12	63	16	25	0	4	9
Manzano other	I	2008	34	1	8.5	64	6	9	0	2	4
Marjanović_AGATE	C	2021	35	2	14	218	64	29	6	1	27
Marjanović_AGATE ETT	I	2021	35	2	14	216	73	34	5	3	21
Martin	C	1993	36	1	9.7	61	4	7	2	0	1
Martin HME	I	1993	36	1	9.7	56	0	0	0	1	0
Miano	C	2009	37	1	4	377	31	8	6	3	3
Miano Gastric	I	2009	37	1	4	457	5	1	0	0	0
Morrow	C	2010	38	2	14.6	73	33	45	6	2	14
Morrow probiotic	I	2010	38	2	14.8	73	17	23	0	3	8
Nseir	C	2011	39	1	10	61	16	26	2	2	3
Nseir ETT	I	2011	39	1	12	61	6	10	0	1	1
Pickworth	C	1993	40	2	7.4	39	6	15	.	.	0
Pickworth Gastric	I	1993	40	2	7.2	44	5	11	1	.	1
Pneumatikos	C	2006	41	1	16	40	11	28	1	1	4
Pneumatikos other	I	2006	41	1	15	39	6	15	0	1	2
Prod’hom	C	1994	42	2	5.6	81	18	22	4	0	5
Prod’hom Gastric	I	1994	42	2	5.2	81	18	22	4	0	5
Prod’hom	C	1994	42	2	5.1	80	22	28	1	1	4
Prod’hom Gastric	I	1994	42	2	5.2	83	11	13	1	0	2
Reigneir	C	2013	43	2	10	222	35	16	9	.	17
Reigneir other	I	2013	43	2	10	227	38	17	12	.	10
Rongrungruang	C	2015	44	1	8	75	22	29	3	6	.
Rongrungruang probiotic	I	2015	44	1	8	75	18	24	2	8	.
Rumbak	C	2004	45	1	5	60	15	25	5	.	5
Rumbak other	I	2004	45	1	16	60	3	5	1	.	1
Ryan	C	1993	46	2	5.6	58	8	14	1	0	2
Ryan Gastric	I	1993	46	2	5.1	56	7	13	2	1	1
Smulders	C	2002	47	1	14.2	75	12	16	3	.	3
Smulders SSD	I	2002	47	1	11.9	75	3	4	1	.	1
Somberg	C	2008	48	2	16	167	16	10	3	1	2
Somberg Gastric	I	2008	48	2	15	35	3	9	0	0	0
Staudinger	C	2010	49	1	14	75	17	23	5	.	2
Staudinger Position	I	2010	49	1	8	75	8	11	3	.	2
Thomachot	C	1998	50	1	12	66	21	32	3	1	7
Thomachot HME	I	1998	50	1	12	70	26	37	2	1	8
Thomachot	C	1999	51	1	11	77	24	31	1	0	8
Thomachot HME	I	1999	51	1	12	63	21	33	2	0	7
Thomachot	C	2002	52	1	9	84	22	26	2	0	7
Thomachot HME	I	2002	52	1	9	71	10	14	0	1	5
Valencia	C	2007	53	1	13	69	10	14	1	0	2
Valencia Position	I	2007	53	1	13	73	11	15	3	1	2
Walaszek	C	2017	54	1	5	804	86	11	5	39	4
Walaszek SSD	I	2017	54	1	5	1003	43	4	10	22	2
Zeng	C	2016	55	1	22	117	59	50	19	14	16
Zeng probiotic	I	2016	55	1	18	118	43	36	13	10	12
**Antiseptic**											
Caruso	C	2009	56	2	17	132	31	23	9	5	5
Caruso Saline	I	2009	56	2	17	130	14	11	7	1	1
Fourrier	C	2000	57	2	24	28	14	50	4	2	3
Fourrier Chlx	I	2000	57	2	18	30	5	17	1	2	0
Fourrier	C	2005	58	2	13	114	12	11	5	0	2
Fourrier Chlx	I	2005	58	2	14	114	13	11	6	1	1
Koeman	C	2006	59	2	13	130	23	18	4	.	5
Koeman-Chlx	I	2006	59	2	14	127	13	10	0	.	2
Koeman-Chlx + C	I	2006	59	2	13	128	16	13	2	.	5
Kollef	C	2006	60	2	14	347	63	18	9	2	25
Kollef Iseganin	I	2006	60	2	14	362	80	22	8	2	17
Sebastian	C	2012	61	2	8	45	14	31	3	8	0
Sebastian	I	2012	61	2	8	41	12	29	3	6	1
Seguin—SC	C	2006	62	2	14	31	12	39	1	.	7
Seguin—CC	I	2006	62	2	19	31	13	42	0	.	7
Seguin-PVI	I	2006	62	2	15	36	3	8	0	.	3
Seguin	C	2014	63	2	16	72	20	28	1	.	11
Seguin-PVI	I	2014	63	2	15	78	24	31	3	.	14
**Antibiotic**											
Ferrer	C	1994	64	2	7.5	50	11	22	4	0	2
Ferrer TAP + PPAP	I	1994	64	2	7.5	51	7	14	1	0	3
Laggner	C	1994	65	1	30	34	4	12	1	.	0
Laggner TAP	I	1994	65	1	24.9	33	1	3	0	.	0
Abele-Horn	C	1997	66	1	22	30	23	77	3	0	5
Abele-Horn TAP + PPAP	I	1997	66	1	18	58	13	22	2	0	9
Bergmans CC	C	2001	67	2	15	78	24	31	8	.	6
Bergmans TAP	I	2001	67	2	13	87	9	10	3	.	3
Blair	C	1991	68	2	5	130	38	29	9	.	7
Blair TAP + PPAP	I	1991	68	2	5	126	12	10	1	.	1
Bouza	C	2013	69	2	12	38	8	21	3	.	4
Bouza PPAP	I	2013	69	2	10	40	7	18	3	.	2
Claridge	C	2007	70	1	24	52	24	46	9	.	10
Claridge PPAP	I	2007	70	1	21	53	21	40	7	.	10
Dahyot_Fizelie	C	2024	71	2	30	157	58	37	1	.	27
Dahyot_Fizelie PPAP	I	2024	71	2	26	162	35	22	1	.	12
Georges	C	1994	72	1	30	33	15	45	.	0	1
Georges TAP	I	1994	72	1	30	31	4	13	.	1	0
Jacobs	C	1992	73	1	10	43	4	9	0	0	0
Jacobs TAP + PPAP	I	1992	73	1	9	36	0	0	0	0	0
Karvouniaris	C	2015	74	1	13	84	25	30	.	11	4
Karvouniaris TAP	I	2015	74	1	16	84	14	17	.	2	5
Klastersky	C	1974	75	1	19.9	42	17	40	4	.	2
Klastersky TAP	I	1974	75	1	14.7	43	5	12	0	.	0
Korinek	C	1993	76	2	26.6	95	37	39	3	1	16
Korinek TAP	I	1993	76	2	25.4	96	20	21	0	1	9
Nouira	C	2001	77	2	14.5	46	10	22	5	5	0
Nouira PPAP	I	2001	77	2	9.4	47	3	6	0	.	0
Palomar	C	1997	78	2	8.1	46	14	30	1	2	3
Palomar	C	1997	78	2	6.4	42	21	50	6	0	8
Palomar TAP + PPAP	I	1997	78	2	10.8	41	7	17	1	0	5
Pneumatikos	C	2002	79	1	23	30	16	53	1	1	.
Pneumatikos TAP	I	2002	79	1	16	31	5	16	0	1	.
Quinio	C	1996	80	2	15.7	72	38	53	12	.	16
Quinio TAP	I	1996	80	2	16	76	19	25	5	.	9
Reizine	C	2019	81	2	14	149	88	59	4	3	54
Reizine TAP	I	2019	81	2	13	146	57	39	3	1	36
Rocha	C	1992	82	2	8	54	25	46	8	4	15
Rocha TAP + PPAP	I	1992	82	2	8	47	7	15	1	1	5
Sanchez-Garcia	C	1998	83	2	13	140	60	43	.	.	7
Sanchez-Garcia PPAP	I	1998	83	2	13	131	32	24	.	.	5
Sirvent	C	1997	84	1	16	50	25	50	1	2	11
Sirvent PPAP	I	1997	84	1	13	50	12	24	3	1	3
Stoutenbeek	C	2007	85	2	12	200	100	50	28	23	40
Stoutenbeek TAP + PPAP	I	2007	85	2	13	201	62	31	11	15	18
Verwaest	C	1997	86	2	18.9	185	40	22	7	4	9
Verwaest TAP	C	1997	86	2	18.9	200	31	16	7	4	9
Verwaest TAP	I	1997	86	2	17.3	193	22	11	2	4	6
Wiener	C	1995	87	2	11.2	31	8	26	0	0	4
Wiener TAP + PPAP	I	1995	87	2	11.4	30	8	27	2	1	1
Winter CC	C	1992	88	2	8	92	17	18	8	2	1
Winter TAP + PPAP	I	1992	88	2	6.4	91	3	3	3	0	0

Abbreviations: LOS = group mean or median length of stay; C/I = control/intervention groups; QS = quality score; VAP = ventilator-associated pneumonia; ‘.’ = count not reported. Additional data for study 86 obtained from [89]. Studies 2, 3, 12, 17, 37, 40, 42, 46, and 48 were found in [90]; studies 13, 19, 38, 44, and 55 were found in [91]; study 9 was found in [92]; study 21 was found in [93]; studies 11, 18, 23, 30, 36, and 52 were found in [94]; studies 28 and 29 identified in [95]; no eligible studies were found in [96,97]; studies 56, 57, 58, 59, 61, 62, and 63 were found in [98,99]; studies 64, 65, 66, 67, 68, 72, 76, 78, 79, 80, 82, 83, 85, 86, 87, and 88 were found in [100,101]. Intervention categories: ETT = endotracheal tube; HME = heat and moisture exchange; SSD = subglottic secretion drainage; Chlx = chlorhexidine; PVI = povidine iodine; TAP = topical antibiotic prophylaxis; PPAP = protocolized parenteral antibiotic prophylaxis; CC = Usual Care Control; SC = saline control.

**Table 2:** Characteristics of studies ^a^.

	Non-Antimicrobial	Antiseptic	Antibiotic
Study characteristics			
Ref.	[1,2,3,4,5,6,7,8,9,10,11,12,13,14,15,16,17,18,19,20,21,22,23,24,25,26,27,28,29,30,31,32,33,34,35,36,37,38,39,40,41,42,43,44,45,46,47,48,49,50,51,52,53,54,55]	[56,57,58,59,60,61,62,63]	[64,65,66,67,68,69,70,71,72,73,74,75,76,77,78,79,80,81,82,83,84,85,86,87,88]
Number of studies	55	8	25
Origin from systematic review ^b^	18	8	17
North American ICUs ^c^	13	1	2
Control group PPAP ^d^	0	0	2
Group mean age; (mean)	55.4	48.5	50.1
Group mean age; (95% CI)	52.3 to 58.4	42 to 54.5	46.2 to 54
Study publication year (median)	2006	2006	1997
Study publication year (range)	1987–2022	2000–2014	1974–2024
Group characteristics			
Types of groups			
CC	56	9	27
Intervention	56	10	28
Numbers of patients per group; (median) ^e^	83	72	52
Numbers of patients per group; (IQR) ^e^	61 to 166	31 to 130	42 to 130
Group mean LOS; (mean)	12.9	11.4	15.9
Group mean LOS; (95% CI)	11.3 to 14.4	8.3 to 14.5	14 to 17.9

^a^ Note, several studies had more than one control and/or intervention group. Hence the number of groups does not equal the number of studies. ^b^ Studies that were sourced from 12 systematic reviews. ^c^ Study originating from an ICU in Canada of the United States of America. ^d^ Use of protocolized parenteral antibiotic prophylaxis (PPAP) for control group patients. ^e^ Data median and inter-quartile range (IQR).

**Table 3:** Contrast-based versus arms-based analyses ^a^.

	Non-Antimicrobial		Antiseptic		Antibiotic	
	summary	95% CI (n)	summary	95% CI (n)	summary	95% CI (n)
Overall VAP ^b^						
Contrast-based odds ratio	0.73	0.61 to 0.86 (56)	0.53	0.34 to 0.81 (11)	0.39	0.33 to 0.46 (28)
Arms-based (SROC)						
DOR	0.71	0.6 to 0.84 (56)	0.52	0.34 to 0.8 (11)	0.39	0.32 to 0.47 (28)
control (mean %)	21	18 to 23	25	19 to 34	34	28 to 39
intervention (mean %)	15	13 to 19	16	11 to 22	16	13 to 20
*Pseudomonas* VAP ^c^						
Contrast-based odds ratio	0.8	0.67 to 0.95 (54)	0.65	0.42 to 1.01 (11)	0.43	0.31 to 0.6 (25)
Arms-based (SROC)						
DOR	0.77	0.62 to 0.96 (54)	0.59	0.36 to 0.98 (11)	0.41	0.28 to 0.59 (25)
control (mean %)	3.5	2.7 to 4.5	2.8	1.6 to 4.9	5.8	4.2 to 8.1
intervention (mean %)	2.7	1.9 to 3.5	1.7	0.8 to 3.5	2.4	1.6 to 3.6
*Acinetobacter* VAP ^d^						
Contrast-based odds ratio	0.85	0.62 to 1.16 (40)	0.73	0.34 to 1.54 (7)	0.6	0.38 to 0.93 (17)
Arms-based (SROC)						
DOR	NA		NA		0.61	0.31 to 1.2 (17)
control (mean %)					2.3	1.3 to 4.2
intervention (mean %)					1.4	0.7 to 2.7
*S. aureus* VAP ^e^						
Contrast-based odds ratio	0.79	0.65 to 0.95 (53)	0.48	0.31 to 0.74 (11)	0.55	0.45 to 0.69 (27)
Arms-based (SROC)						
DOR	0.65	0.51 to 0.83 (53)	0.43	0.29 to 0.64 (11)	0.58	0.43 to 0.79 (27)
control (mean %)	4.7	3.7 to 5.9	5.6	3.3 to 9.7	7.6	4.9 to 11.4
intervention (mean %)	3.1	2.2 to 4.3	2.4	1.1 to 5.3	4.5	2.9 to 7.1

^a^ Abbreviations: CI = confidence interval; DOR = diagnostic odds ratio. ^b^ The SROC analyses for overall VAP are displayed as Figure 2a–c. ^c^ The SROC analyses for *Pseudomonas* VAP are displayed as Figure 3a–c. ^d^ These estimates from were not available (= NA) as the SROC model failed to converge in the analysis of the non-antimicrobial and antiseptic groups for *Acinetobacter* VAP. ^e^ The SROC analyses for *S. aureus* VAP are displayed as Figure 4a–c.

**Table 4:** Arms-based meta-regression of VAP incidence for control and intervention groups ^a^.

	Unadjusted Model			Model Adjusted for Mean LOS ^b^		
	Coefficient	95% CI	*p*	Coefficient	95% CI	*p*
Overall VAP						
Constant	−1.34	−1.53 to −1.15	<0.01	−2.64	−3.38 to −1.91	<0.01
Non-antimicrobial Intervention	−0.32	−0.57 to −0.06	0.02	−0.31	−0.56 to −0.07	0.01
Antiseptic Control	0.3	−0.23 to 0.82	0.27	0.12	−0.39 to 0.63	0.64
Antiseptic Intervention	−0.65	−1.14 to −0.16	0.01	−0.41	−0.94 to −0.11	0.12
Antibiotic Control	0.67	0.33 to 1.01	<0.01	0.47	0.14 to 0.81	0.01
Antibiotic Intervention	−0.16	−0.5 to 0.19	0.37	−0.3	−0.64 to 0.08	0.13
Year of Publication ^c^				0.01	−0.01 to 0.02	0.11
Majority Quality Score ^d^				0.03	−0.21 to 0.22	0.83
LOS (log transformed)				0.54	0.31 to 0.77	<0.01
*Pseudomonas* VAP						
Constant	−3.13	−3.36 to −2.89	<0.01	−3.94	−4.8 to −3.1	<0.01
Non-antimicrobial Intervention	−0.34	−0.68 to 0.00	0.05	−0.28	−0.58 to −0.02	0.07
Antiseptic Control	0.02	−0.63 to 0.66	0.96	0.03	−0.58 to 0.64	0.93
Antiseptic Intervention	−0.67	−1.28 to −0.06	0.03	−0.3	−0.94 to 0.34	0.36
Antibiotic Control	0.5	0.08 to 0.88	0.01	0.42	0.03 to 0.82	0.04
Antibiotic Intervention	−0.39	−0.84 to 0.06	0.09	−0.52	−0.95 to −0.09	0.02
Year of Publication ^c^				−0.01	−0.02 to 0.01	0.16
Majority Quality Score ^d^				−0.07	−0.36 to 0.23	0.66
LOS (log transformed)				0.42	0.15 to 0.69	<0.01
*Acinetobacter* VAP						
Constant	−4.01	−4.4 to −3.62	<0.01	−2.56	−4.03 to −1.08	<0.01
Non-antimicrobial Intervention	−0.02	−0.57 to 0.54	0.95	−0.04	−0.55 to 0.46	0.86
Antiseptic Control	0.66	−0.46 to 1.79	0.25	1.05	−0.03 to 2.13	0.06
Antiseptic Intervention	−0.43	−1.44 to 0.59	0.41	0.51	−0.61 to 1.63	0.37
Antibiotic Control	0.68	0.16 to 1.46	0.01	1.12	0.42 to 1.82	<0.001
Antibiotic Intervention	0.06	−0.66 to 0.78	0.87	0.32	−0.41 to 1.06	0.39
Year of Publication ^c^				0.02	−0.01 to 0.04	0.1
Majority Quality Score ^d^				−0.71	−1.23 to −0.2	0.01
LOS (log transformed)				−0.17	−0.65 to 0.30	0.47
*S. aureus* VAP						
Constant	−2.91	−3.2 to −2.65	<0.01	−4.96	−5.98 to −3.95	<0.01
Non-antimicrobial Intervention	−0.36	−0.72 to −0.01	0.05	−0.36	−0.71 to −0.02	0.04
Antiseptic Control	0.43	−0.25 to 1.1	0.22	0.05	−0.64 to 0.73	0.89
Antiseptic Intervention	−0.62	−1.27 to 0.04	0.06	−0.76	−1.49 to −0.03	0.04
Antibiotic Control	0.57	0.12 to 1.02	0.01	0.22	−0.23 to 0.68	0.33
Antibiotic Intervention	0.28	−0.16 to 0.73	0.21	−0.18	−0.63 to 0.27	0.44
Year of Publication ^c^				0.01	−0.01 to 0.02	0.84
Majority Quality Score ^d^				0.34	0.01 to 0.66	0.04
LOS (log transformed)				0.71	0.39 to 1.03	<0.01

^a^. Abbreviations: CI = confidence interval; LOS = length of stay. ^b^. Findings obtained from meta-regression limited to RCCTs sourced from Cochrane reviews were similar. ^c^. Year of publication centered on 1980. ^d^. Majority quality scores were based on the score as rated in the original Cochrane review source documents.

**Table 5:** Comparison with previous antimicrobial-based prevention effect size estimates.

	Antiseptic-Based				Antibiotic-Based			
	Coefficient	95% CI;	n ^a^	Ref.	Coefficient	95% CI;	n ^a^	Ref.
VAP or RTI (Overall)								
RTI	RR: 0.76	0.62 to 0.91	18	[98,99]	OR: 0.28	0.2 to 0.38	16	[100]
VAP	OR: 0.68	0.53 to 0.87	21	[122]	RR: 0.43	0.35 to 0.53	17	[101]
NP/RTI	RR: 0.73	0.58 to 0.92	16	[126]	RR: 0.44	0.36 to 0.54	22	[118]
VAP	OR: 0.53	0.34 to 0.81	11	This study	OR: 0.39	0.33 to 0.46	28	This study
VAP	DOR: 0.52	0.34 to 0.8	11	This study	DOR: 0.39	0.32 to 0.47	28	This study

Abbreviations: OR = odds ratio; RR = risk ratio; DOR = diagnostic odds ratio; 95% CI = 95% confidence interval; RTI = respiratory tract infection; NP = nosocomial pneumonia; VAP = ventilator-associated pneumonia; n = number of studies. ^a^. n is number of studies.

## Data Availability

The original contributions presented in this study are included in the article. Further inquiries can be directed to the corresponding author(s).

## Abbreviations

The following abbreviations are used in this manuscript:

ICU	Intensive Care Unit
IQR	Inter-Quartile Range
LOS	Length Of Stay
MV	Mechanical Ventilation
RCCT	Randomized Concurrent Controlled Trial
SROC	Summary Receiver Operator Characteristic
TAP	Topical Antibiotic Prophylaxis
VAP	Ventilator-Associated Pneumonia
